# Perceived barriers to early diagnosis of breast Cancer in south and southwestern Ethiopia: a qualitative study

**DOI:** 10.1186/s12905-020-00909-7

**Published:** 2020-02-27

**Authors:** Sefonias Getachew, Aragaw Tesfaw, Mirgissa Kaba, Andreas Wienke, Lesley Taylor, Eva J. Kantelhardt, Adamu Addissie

**Affiliations:** 1 Department of Preventive Medicine, School of Public Health, Addis Ababa University, Addis Ababa, Ethiopia; 2 Institute of Medical Epidemiology, Biometrics and Informatics, Martin-Luther-University, Halle, Germany; 3 Department Public Health, College of Health Science, Debre Tabor University, Debre Tabor, Ethiopia; 4 City of Hope National Medical Center, Duarte, Los Angeles, California USA; 5 Department of Gynaecology, Martin-Luther-University, Halle, Germany

**Keywords:** Breast cancer, Early diagnosis, Barriers, Ethiopia

## Abstract

**Background:**

Early diagnosis is a key determinant of breast cancer prognosis and survival. More than half of breast cancer cases are diagnosed at an advanced stage in Ethiopia, and the barriers to early diagnosis in this country are not well understood. We aimed to identify the perceived barriers to early diagnosis of breast cancer from the perspective of patients and health care providers in south and southwestern Ethiopia.

**Methods:**

A qualitative study was conducted from March to April 2018 using in-depth interviews of breast cancer patients and breast cancer health care providers from six public hospitals located in urban and rural areas of south and southwestern Ethiopia. All participants provided verbal consent before participating. A thematic analysis was performed using Open Code 4.02.

**Results:**

Twelve breast cancer patients and thirteen health care providers were included in the study. Patient and health-system related barriers to early diagnosis of breast cancer were identified. Patient-related barriers were lack of knowledge and awareness of breast cancer, belief in traditional medicine and religious practices for treatment, and lack of social and financial support to seek care at a medical facility. Health-system related barriers were misdiagnosis of breast cancer, long distance to referral facilities, high cost of diagnostic services, long waiting time for diagnostic tests, and lack of screening and diagnostic tests in local facilities.

**Conclusions:**

Early diagnosis of breast cancer is affected by multiple barriers in south and southwestern Ethiopia. Awareness campaigns and education about the disease, prevention, and early detection are needed to increase early diagnosis of breast cancer. Opportunities exist to improve early diagnosis and timely treatment in rural areas.

## Background

Breast cancer is the most prevalent cancer in African women, responsible for one in four diagnosed cancers and one in five cancer deaths [1]. In Ethiopia, in the year 2015, it was estimated that the prevalence of breast cancer case is 13,987 with a crude incidence rate of 28.2 per 100,000 and it accounts for 33% of all cancer cases among women [2]. The global cancer data (GLOBOCAN) also estimated in 2018 for Ethiopia the age standardized incidence and mortality rate per 100, 000 among all ages to be 41.2 and 22.9 [1]. If breast cancer is identified at an early stage (I-II), it has a high chance of cure and is more likely to respond to treatment than if diagnosed at a later stage (III-IV) [3]. Delays in diagnosis lead to advanced stage presentation and poor clinical outcomes [4, 5]. Most breast cancer patients in low-income countries such as Ethiopia experience very long delays, are diagnosed at advanced stages, and have low survival rates [6, 7].

Previous studies have identified several patient and health system -related barriers to early diagnosis of breast cancer in Africa but the barriers vary from region to region [6–9]. African women delay presenting to health facilities mostly due to lack of information or knowledge about initial signs and symptoms, poor quality of health facilities, belief in traditional medicine in favour of seeking medical care, lack of trust, and a lack of access to health care [10, 11]. Belief in traditional medicine, religious beliefs, economic hardship, poor provider knowledge, and misdiagnosis were major reasons for a delay in diagnosis in Malawi [12].While a low education level was also associated with a patient delay in Rwanda [13]. Health care provider factors that contribute to delayed diagnosis in Africa include incomplete patient examination, inappropriate use of diagnostic tests, misinterpretation of test results, and misdiagnosis [14]. Less comprehensive health insurance coverage and false negative diagnostic tests are also common health-system related factors [15].

Breast cancer awareness and knowledge about the benefits of early detection and diagnosis are poor in most sub-Saharan African countries including in Ethiopia, and consequently, advanced stage presentation is a common problem in the region [16–19]; and women living in rural areas present at a later stage of breast cancer than women living in urban areas [20, 21].

In Ethiopia the national cancer control plan was launched in 2016 [22] and as result health care providers’ capacity and training are expanding. Screening for breast cancer is recommended for women over the age of 18 years. However, organized screening modalities are still not in place at the facility and community levels. Despite lack of specific breast cancer screening and care guideline in the country, clinical breast examination services are provided in some facilities.

Thus, given the clinical significance associated with delayed diagnosis and the paucity of understanding related to the reasons for delayed breast cancer diagnosis in Ethiopia, we examined the perceived barriers to early diagnosis of breast cancer in Ethiopia from the perspective of patients and health-care providers.

## Methods

### Study approach and setting

A qualitative in-depth interview method was used to explore perceptions of barriers related to early diagnosis of breast cancer among breast cancer patients and health-care providers. The study was conducted from March to April 2018 and included six public hospitals in south and southwestern Ethiopia (Durame General Hospital, Attat Hospital, Dubo St. Mary Hospital, Wolliso St. Lukas Hospital, Butajira General Hospital, and Hawassa Comprehensive Referral Hospital).

### Participants and recruitment

The participants in this study were newly diagnosed female breast cancer patients (age > 18 years old) and health-care providers (surgeons, oncologist, and nurses) who were involved in the diagnosis and management of breast cancer for ≥ 1 year. Breast cancer patients receive follow up are in surgery departments only as there are no oncology clinics available at those sites. Therefore, health care providers were invited from those surgery departments. The patients were recruited through discussions with surgeons and nurses from surgical department and oncology units on the basis of their lived experiences and understanding of the study.

The hospitals were selected because they serve mostly rural populations and have experience with treatment services for breast cancer patients. Hospitals were approached and the hospital leadership linked the research team to the providers and patients. The research team gave all the hospitals, health-care providers, and patients a description of the study. The research team gave all the hospitals, health-care providers, and patients a description of the study including the objectives, the benefit expected from the study findings and the fact that participation in interviews was voluntary.

### Ethical considerations

Ethical clearance was approved from the Research and Ethical Committee (REC) of School of Public Health, and the Institutional Review Board (IRB) of College of Health Science Addis Ababa University. Verbal consent was obtained from each of the patients in the study and written consent is taken from health care providers. Verbal consent was taken from the patients based as the rapid ethnographic assessment (REA) conducted to inform the informed consent process revealed that majority are not able to read and write in addition to major cultural concerns on signature. This procedure was approved by the Institutional Review Board of Addis Ababa University mentioned above. In addition, permissions were obtained from the selected hospitals prior to data collection. The objectives and importance of the study were explained to the participants before the interview. Participants were notified that their participation was voluntary and that they could withdraw at any time during the interview process. All information provided by the participants was de-identified to maintain participant privacy.

### Data collection

In-depth interviews were conducted for each participant using a semi-structured interview guide to balance openness and focus during the interviews. The guide was developed by the research team following a thorough literature review and initial key informant interviews. It was first developed in English and then translated to the local language (*Amharic*) to facilitate communication with participants. Interview themes/questions focused on healthcare provider experiences with breast cancer early detection methods and potential reasons for late diagnosis of breast cancer or perceived lack of early diagnosis, and on patient experiences with what was felt when a breast change was first noticed and reasons for visiting a health facility after the disease had advanced (for the complete interview guide see Supplementary File 1)**.** All interviews were conducted within the hospital setting during the day; and lasted 40–60 min. All interviews were tape-recorded and transcribed verbatim and translated into English. All information was de-identified under transcription. Transcripts and translations were cross-checked for accuracy and consistency. Appropriate written notes were also taken in the event the audio recorder could not capture content during the interviews. The interview process was continued until the data reached saturation, defined as that point when answers were no longer providing new/additional information to the research question and recurrent patterns became evident in the patients’ narratives.

### Data analysis

Translated notes were read and re-read by two independent coders who had qualitative research experience. The data were then organized by coding or dividing the text into meaningful elements using Open Code version 4.02 software. The coding process continued until all the data were exhausted. The coders identified themes and sub-themes from the data and thematic analysis was used to identify themes and sub-themes by grouping related codes.

The analysis was mainly performed by the author and all co-authors reviewed and approved the different scripts and developed themes and sub themes.

## Results

### Socio-demographic characteristics

Twenty five patients and health care providers participated; 12 breast cancer patients and 13 healthcare providers. Patient age ranged from 26 to 65 years (Table 1).

**Table 1:** Socio demographic profile of breast cancer patients involved in the interviews

Characteristics	Frequency	Percentage
**Age**		
≤ 30	1	8.3
31–40	8	66.8
41–50	1	8.3
51–60	1	8.3
≥ 61	1	8.3
**Residence**		
Urban	4	33.3
Rural	8	66.7
**Marital status**		
Married	9	75.0
Widowed	3	25.0
**Educational status**		
Illiterate	5	41.7
Primary school	3	25.0
Secondary school	1	8.3
Diploma and above	3	25.0
**Occupational status**		
House wife	2	16.7
Farmer	6	50.0
Merchant	1	8.3
Government employ	3	25.0

Health care providers included an oncologist, gynaecologist and obstetrician, general surgeons, public health officers, and nurses who were involved in diagnosis, treatment and management of breast cancer patients (Table 2).

**Table 2:** Socio-demographic profile of Health care professionals involved in the interviews

Characteristics	Frequency	Percentage
**Age**		
20–30	5	38.5
31–40	5	38.5
41–50	2	15.3
> 51	1	7.7
**Educational status**		
Diploma level	3	23.0
Degree level	5	38.5
Specialization level	5	38.5
**Profession**		
Nurse	6	46.3
Public health officer	2	15.3
General Surgeon	3	23.0
Gynecologist	1	7.7
Clinical Oncologist	1	7.7

### Themes and sub-themes (categories) identified

Two main themes emerged from the narrations of the participants regarding the barriers to early diagnosis of breast cancer - patient- and health system-related barriers. Five sub-themes were identified. Within the theme of patient-related barriers, three sub-themes were identified - lack of awareness and knowledge about breast cancer, beliefs in traditional and religious treatments, and lack of financial and social support. Within the theme of health system-related barriers, two sub-themes were identified - services rendered by healthcare providers and the functioning of the health care facilities.

### Patient related-barriers

Lack of knowledge and awareness about breast cancer.

Almost all patients described a general lack of awareness and knowledge about risk factors, signs and symptoms of breast cancer as well as a general lack of community activity and attention given to early detection of breast cancer. Most patient participants were unaware of breast cancer before diagnosis and had limited knowledge about the disease, and this had great impact on when they sought medical attention. Most sought medical intervention once the disease was advanced. Most patient participants explained that they detected abnormalities on their breasts accidentally when they were in bathroom or when undressing before bed. None had a history of breast self-examination or clinical check-ups.

***“****I never heard about breast cancer before. I saw the swelling four years back but I did not inform to anyone in the family since it was painless but later becomes large and produce discharge.” (Patient, Age range 41–50 yrs old).*

***“****I did not check my breast before. I saw the swelling accidentally. I did not go to health facility for check-up of my breast.” (Patient, Age range 21–30 yrs old).*

Patients’ perceptions that their initial symptoms were harmless delayed them seeking medical care. Many failed to seek early medical care unless the symptoms interfered with their day-to-day activities. Some did not give attention to early symptoms.

*“I saw the swelling on my breast 4 years ago. But I was not going to any health facility since I was healthy for a long time and it was painless.”(Patient, Age range 31–40 yrs old).*

Patients also explained that the painless nature of a swelling or mass led them to perceive the breast change as a common and self-limiting problem, and this resulted in delays seeking medical care. Many patients explained they did not initially think their symptoms could be cancer and all patients sought medical care at least 12 months after they first noticed symptoms. Patients from rural areas described delays in seeking healthcare due to distance, transportation challenges, cost of care, but mostly due to lack of information related to the disease.

Beliefs in traditional medicine and religious practice for treatments.

All patients interviewed delayed seeking medical care in favour of using traditional and spiritual treatments (holy water) because there is a preference to go traditional healers as the first choice of treatment rather than to heath care facilities; many also believed that swelling could be healed or treated by applying herbal medications. Patients stated that limited knowledge led them to perceive their illness with the same traditional beliefs pervasive in many villages.

*“I was using traditional treatment like onion, Tena Adam and other leaves. I thought it was the disease given from God.*” *(Patient, Age range 51–60 yrs old).*

*“I went to traditional healer and I took herbal medication for a long time since I thought it as ‘Bigunji’ (local name to swelling with pus) and continued to use the traditional treatment. Finally I came to hospital when the disease becomes sever and sever.*” (*Patient, Age range 41–50 yrs old).*

The patient interviews further revealed different beliefs that contributed to delays in seeking medical care for breast cancer. Losing a breast (mastectomy) was described as taboo. The fear expressed was that if a woman loses her breast due to surgery, she might die, could not give birth, or she would be divorced by her husband. As a result, most women hid their problem and sought medical care as a final option only after trying all traditional or cultural treatment approaches. Again, patients shared that most of people are not aware of the disease or its treatment, and therefore don’t advise women to go health facilities. Swelling in the breast is not perceived as a treatable medical problem, but rather as “God’s punishment”. This ailment commonly called “Meksefit” in the community is locally believed as a disease related to demonic/ witchcraft.

***“****My community does not support the medical treatment related breast cancer since they perceived that……..if it is touched by scissor or syringe it will spread and kill the patient.” (Patient, Age range 21–30 yrs old).*

*“I was not thinking that cancer has any treatment in the hospital since my community perceived that cancer is killer and does not have any treatment in the hospital. But we believe on herbal medication and holy water.” (Patient, Age range 41–50 yrs old).*

### Lack of financial and social support

Many patients explained they did not have financial resources to afford the treatment and transportation costs; thus contributing to delays in care and a barrier to early diagnosis. Those individuals with economic means were better positioned to get early diagnosis and treatment. Patients described borrowing money from a neighbor, friend, or family to get medical care, but explained how this was not easy in their communities where everyone had limited economic recourses. Patients also felt their family responsibilities and a general lack of social and family support prevented them from seeking early medical care; and even after seeking medical care almost all patients face economic hardship with little money to pay for medical costs. To amass money for care patients engaged in borrowing, begging on streets, or selling their land, cattle, or other property; and during that time interval the disease is untreated and progresses.

*“I have family responsibility since my husband is died. My breast problem starts before two years. But I could not come early to health facility because I did not have money for transport and treatment service requested, there is no anyone who supports me.”(Patient, Age range 51–60 yrs old).*

*“I had no money for medication and transportation since my husband was died and all the family responsibility is on me. Even now I was not paid the money for surgery. I came to this hospital after selling my farm land and borrowing some money from my families.”(Patient, Age range 31–40 yrs old).*

### Health system related barriers

#### Health care provider’s perception related barriers

Similar to patient accounts, providers also described poor breast self-awareness and knowledge regarding importance of recognizing breast changes, and early signs and symptoms of breast cancer.

*“Majority of the patients are coming late after the disease is advanced. This is because lack of awareness and knowledge about the disease since they did not consider the initial signs and symptoms as serious.” (Gynaecologist, Age range 51–60 yrs old).*

***“****Most of the patients came at advanced stage (Above stage III) and this is due to lack of awareness about the disease. Their awareness about breast self-examination and early clinical check-up is nil”. (General Surgeon, Age range 21–30 yrs old).*

Healthcare providers also stated that most women coming from rural areas present at advanced stage disease compared to urban patients. Providers described a perception that lack of access to information among rural women may have to do with lower level of education, literacy rate, working in the home.

*“……since there is no information access either through magazines, newspapers or media, the awareness of the rural people is very low. Peoples from the urban areas relatively come early as they feel any changes on their breast.”(General Surgeon, Age range 31–40 yrs old).*

The providers added that most patients go to the hospital as a last option after the cancer is advanced and/or is metastatic. They stated most patients perceive cancer as a disease related traditional or cultural things. As a result, patients would take herbal medications or holy water remedies. Also, providers described fear of receiving care in health facilities. For example, providers described a belief that if someone were to go to a health facility and get an injection, the needle would hide the disease; it would spread inside the body, and then kill the patient.

***“****There are a lot of patients who went to their home without getting definitive diagnosis due to their inability to pay for transportation and diagnosis when they are referred.” (Nurse, Age range 21–30 yrs old).*

“*I can say almost all patients are used herbal medications before coming to hospital. Some patients also spend most of the time going to holy water in orthodox religion and praying in Protestants since they consider it as a cultural disease”. (Nurse, Age range 21–30 yrs old).*

Many patients experienced delays in transitions of care due to poor provider knowledge and misdiagnosis. Patients are often given analgesics or other treatments for several months at primary care facilities before receiving an appropriate referral to a facility for breast cancer care. They describe delays in getting the right diagnosis and how the providers contribute to delays in care. Misdiagnosis and delays in referring patients early to the regional diagnostic hospital were the most frequently mentioned problem leading to patient’s presenting with late stage disease. Almost all patients described a history of misdiagnosis during their initial visits to the health care provider and being placed on some form of prolonged treatment for an incorrect diagnosis.

*“I went to private health facility first and they told me as it is other breast problem and they gave me treatment but I was not improved. Then I went to another private hospital and they said me you have breast TB and they gave me TB treatment and I took for 6 month.” (Patient, Age range 31–40 yrs old).*

“*First I went to the private clinic and the doctor told me that do not worry for this. It is the effect of the contraceptive you used before so it would be lost by itself and he ordered me 6 injections. I was hoping him and I wait a long time but the swelling becomes increase and starts to produce bloody discharge then I came to hospital and diagnosed as cancer.”(Patient, Age range 31–40 yrs old).*

Health care providers also stated that misdiagnosis is a common problem in breast cancer care. This is usually a problem at primary health care facilities since they fail to detect such cases early and refer patients in a timely fashion. Clinical breast examination is not commonly practiced in the facilities unless patients are coming with complaint of breast abnormalities. Patients elaborated on appointment delays, poor attention given to them while in the facilities, inadequate examinations and poor communication between health care providers and patients. These were some of the reasons which led patients not to have additional follow up visits to the health care facilities and instead look to traditional means of treatment.

“*Usually there is misdiagnosis of cases especially among the young’s as a breast lump or fibro adenoma but after sometime the patients may came again with advanced stage breast cancer. I know a woman who was diagnosed with other breast problem but after a long time she come again and diagnosed as breast cancer.” (Patient, Age range 31–40 yrs old).*

**“***The first doctor who has diagnosed me was not good. He said all part of your breast should be removed. He did not reassure me. He made me to worry and frightened then I went to traditional treatment areas but I was not improved of the problem and now the disease is spread to my body.”(Patient, Age range 21–30 yrs old).*

### Health facility related barriers

The patients faced problems with access to health facilities due to long distances of the facilities from their home and high transportation costs. The referral experience was also very poor at health centers and private clinics even in cases when patients presented early for appropriate diagnosis and treatment. All of the patients had a referral history to other health facilities for diagnostic investigations. The most important health facility barrier mentioned by the providers and patients was diagnostic waiting time. They mentioned that the waiting time for getting pathologic lab test results takes usually more than a month, and the absence of the tests in some of the hospitals makes the problem worse. Patients explained they required several visits to health facilities to get their diagnosis. They first went to health centres where drugs were prescribed without proper assessment. When they saw that their situation was not changing as they expected, the patients then went to private clinics. It was after these attempts that the women were then finally referred to the diagnosing hospital. At a minimum, patients visited two health facilities before they got their final diagnosis. The providers similarly explained that the absence of diagnostic tests and treatment options for breast cancer in the hospitals were the main problem contributing to delays in care. Providers described situations when they felt obligated to refer patients to other hospitals for diagnosis and treatments, knowing this could be very expensive for patients and that they quite possibly patients would not be able to afford to go the referral center.

***“****I wait a month to get my laboratory result but I faced many difficulties when they referred me to this hospital because of distance. Since there is no transport access and the medical care costs are so expensive. I came to this hospital last week but they said me there is no bed and I returned back at that time and now I came again. I visited three health facilities one private and two government health facilities before.”(Patient, Age range 31–40 yrs old).*

***“****Our major challenge here is we have no diagnostic tests, standard treatments and a screening tool. So we refer patients to Addis Ababa for pathologic tests and treatments but sometimes they come back without getting the service due to over schedule, so we cannot do anything to them because they have non-operable cancer then they will disappeared or die in their home.” (Surgeon, Age range 31–40 yrs old).*

Patients explained that the absence of screening and health education programs, including skilled professionals, are some of the health system challenges to providing early detection, diagnosis and treatment of breast cancer. Once the patients have already paid for prior (incorrect) treatment, travel, and clinic visits during the referral process, they stated they cannot afford the additional costs of care when they arrive at a facility capable of diagnosing and treatment breast cancer. They mentioned that the surgery itself cost much as 2000 Ethiopian birr, and they also have to consider the cost additional transportation, the hospital bed, diagnostic tests, and medications.

“*………..there is no organized way of giving health education about breast cancer in this hospital since there are no skilled professionals on the area.” (Medical oncologist, Age range 21–30 yrs old).*

**“***Cancer treatment is very, very expensive. I have no word to describe the costs. I finished all my money for laboratory, transportation, bed and other treatments. The cost is very much…. not only for the poor but also for the riches. The government should give attention to it.”(Patient, Age range 31–40 yrs old).*

## Discussion

Early stage of diagnosis is a key determinant factor for survival of breast cancer patients, and delays in diagnosis and advanced stage of presentation are associated with poor clinical outcomes [6, 23]. The barriers to early diagnosis of breast cancer are multifaceted ranging from individual patient level to organizational or facility levels. Barriers also vary from country to country, depending on their economic growth, cultural and religious factors, as well as availability of and accessibility to health infrastructure and man power [10].

Our study identified several socio-demographic, economic, cultural, religious, health facility and health care provider related barriers to early diagnosis of breast cancer in south and southwestern Ethiopia. A major patient related barrier to seeking early medical care for breast cancer was the low access to information about breast cancer in the country. This finding is similar to studies in other Sub-Saharan African countries which have shown that lack of knowledge was a major contributing factor for late presentation [15, 22]. Lack of knowledge about the disease delays the search for early medical care, even when patients may have early signs and symptoms because the patients often ascribe different meaning to these breast changes, and may not recognize them as serious. Findings from our study revealed how patients let the sign and symptoms take their natural course without seeking early medical care. This behavior has underpinnings in traditional or religious practices, which are strong and common in Ethiopia [24]. Most patients often first use traditional medicines and holy water before they seek access to medical care, presenting with late stage disease. Patients’ fear of losing their breast, anxiety about medical procedures, failure to disclose their problems to others also contributed to their delay to seek medical attention.

Being from a rural residence and remote areas also contributes to delays in early diagnosis, similar to other studies conducted in African countries [20, 21]. General lack of access to information through magazines, newspapers and media in rural areas was also described as a contributing factor for patients not seeking early medical attention when they noticed breast abnormalities. Patients who had a painful wound or ulcer on their breast were less likely to delay presentation than patients who had painless lump. It was explained that painless lumps were often considered not serious or self-limiting, and patients would seek medical care only when the symptoms became painful or when they interfered with day to day activities. Our finding is consistent with research from Egypt in which women without any pain were more likely to present at later stage than those having pain as the first symptom [25].

Patients’ diagnostic pathways from initial symptom recognition until arrival at cancer diagnosing centers contributed to their delay in diagnosis and advanced stage of presentation. Women faced health system barriers at all points of the diagnostic pathway, especially during diagnostic investigations and the referral period in which patients endured long journeys to diagnostic centers and then long waiting times to get results. A similar situation was reported in a qualitative study in Aracaju, Brazil, even though it is a different social, economic, cultural and health care context [14].

Patients described several barriers they face to seeking early medical care, including family responsibilities, feeling shyness to check their breasts, and fear to disclose their problems to others. They shared a perception that culturally it is not acceptable for women to have one breast, or to show a breast to another person except the husband. Patients also explained the tendency to relate breast changes to symptoms associated with pregnancy, breastfeeding and contraceptive use, thereby delaying them to seek early medical care until the problem worsened.

In Ethiopia the accessibility and availability of cancer diagnostic centers is very limited. There is only one radiotherapy center in the country and majority of diagnostic centers are located at the capital city of Addis Ababa [26]. As a result majority of patients suffer a lot of difficulties including transportation costs and prolonged waiting times to get the service when they referred to diagnostic facilities. The situation has made it difficult for primary health care providers to effectively provide breast cancer diagnostic and treatment services in the rural setups which further create delays in care. Such limitations are reported elsewhere as impediments of cancer care in primary care settings [27].

Our study has strengths and limitations. This is the first study conducted in south and southwestern Ethiopia to explore health system and patient related barriers to early diagnosis of breast cancer. We consider capturing the perspectives from patients and health care providers a major strength of the study. However, we only used in-depth interviews for data collection with limited number of participants.

## Conclusion

Lack of knowledge and awareness, symptom misinterpretation, preference for traditional and spiritual means of treatment, lack of social and family support, and lack of money for medical care costs and transportation, misdiagnosis of cases, long distance to referral centers and long waiting time for diagnostic tests were the major reasons for delays in diagnosis of breast cancer in our study on perspectives of patients and healthcare providers in South and Southwestern Ethiopia.. Our findings show the need for community awareness and education programs about breast cancer signs, symptoms, and treatment options. Opportunities exist to educate healthcare providers, decrease misdiagnosis, strengthen the referral system, and streamline care with the goal of improving outcomes for patients with breast cancer in Ethiopia.

## Supplementary information

**Additional file 1.**

## Data Availability

The raw data analyzed for the current study is deposited by the authors in the data archives of the Non Communicable Diseases (NCD) Research Working Group of Addis Ababa University. Both the first author and the last author are core members of the team. The de-identified data is available upon reasonable request through the authors, please email to: adamu.addissie@aau.edu.et and safoget@yahoo.com .
