# Supplementary material for: Perceived barriers to early diagnosis of breast Cancer in south and southwestern Ethiopia: a qualitative study
Source: BMC Womens Health. 2020 Feb 27;20:38. doi: 10.1186/s12905-020-00909-7 (PMC7045514; doi:10.1186/s12905-020-00909-7)
Supplement: Supplementary file 1 — Additional file 1. [file 12905_2020_909_MOESM1_ESM.docx]

**Interview guide**

1. **Interview guide for the in-depth interview with patients**
2. Would you please tell me your age, educational status, job, marital status and how long have you lived here, how long you have been diagnosed with breast cancer? No need to mention your name.
3. In your opinion, what do you understand about breast cancer before? Prob. Perception about severity of the disease, preventability, risk factors, clinical features and knowledge and practice of early detection methods
4. What did you feel when you notice changes in your breast? Do you think that it will be cancer? To whom you first told your problem, what solutions you made to manage the abnormality in your breast, did you use traditional treatments, holy water?
5. Did you visit any other health facilities before you came to this hospital?
6. How long did you live with the signs or symptoms before you visit health facility? Where were you go in that period?
7. How do you see the communication with health professionals? Did they listen your problems? Privacy? How they fasten your diagnosis? Do you think they make you late?
8. In your opinion why women present at late stage of breast cancer? Prob. Problems related to the awareness, economic and distance issue, health care providers, health facility related.
9. What is thee reason patients to present to heaalth facility after the disease is advanced?
10. In your opinion, what is the challenge or problems related with the health care system?
11. How the community sees breast cancer? Prob. Perception to the disease, severity, stigma and discrimination, support to patients. Do you think it will affect patients not to seek early medical care?
12. Did you fear to be diagnosed as breast cancer? prob. Fear of losing breast, Discrimination, stigma, community and family perception

Thank you I finished my interview. Would like to express my heartfelt thanks for your voluntary participation in this intervie.

**Part II. Interview guide for the in depth interview with health care providers**

1. Would you please tell me your age, educational status, marital status and how long have you worked here?
2. How do you see the health care providers practice of breast cancer early detection methods in your hospital for at risk groups? (Prob. health education on techniques of breast self-examination, clinical breast examinations)
3. In your opinion, why patients diagnosed at late stage of breast cancer? prob. Reasons related patients, health care provider, health facility, biological characteristics of tumor)
4. In your opinion, what are the reasons for poor early diagnosis of breast cancer among patients? Prob. Health care provider, facility level factors?
5. How do you describe breast cancer patient's economic status? Prob. Can they afford treatment and diagnostic costs, transportation, referral, do you think this may contribute for their late diagnosis?
6. Do you think community awareness, culture and other religious factors contribute for late stage presentation? Prob. Any breast taboos, religious, lack of health education programs to community, perception of the community to the disease)
7. How do you describe breast cancer patients understanding about the disease? prob. Their awareness about the sign and symptoms, risk factors, early detection methods, treatments.
8. Do you have anything to share me which is not raised on the discussion before I end the interview?

I am finished my interview with you. I would like to express my heartfelt thanks for your voluntary participation in this in-depth interview
